# Metasurface Freeform Nanophotonics

**DOI:** 10.1038/s41598-017-01908-9

**Published:** 2017-05-10

**Authors:** Alan Zhan, Shane Colburn, Christopher M. Dodson, Arka Majumdar

**Affiliations:** 1 Department of Physics, University of Washington, Seattle, WA-98195 USA; 2 Department of Electrical Engineering, University of Washington, Seattle, WA-98195 USA

## Abstract

Freeform optics aims to expand the toolkit of optical elements by allowing for more complex phase geometries beyond rotational symmetry. Complex, asymmetric curvatures are employed to enhance the performance of optical components while minimizing their size. Unfortunately, these high curvatures and complex forms are often difficult to manufacture with current technologies, especially at the micron scale. Metasurfaces are planar sub-wavelength structures that can control the phase, amplitude, and polarization of incident light, and can thereby mimic complex geometric curvatures on a flat, wavelength-scale thick surface. We present a methodology for designing analogues of freeform optics using a silicon nitride based metasurface platform for operation at visible wavelengths. We demonstrate a cubic phase plate with a point spread function exhibiting enhanced depth of field over 300 micron along the optical axis with potential for performing metasurface-based white light imaging, and an Alvarez lens with a tunable focal length range of over 2.5 mm corresponding to a change in optical power of ~1600 diopters with 100 micron of total mechanical displacement. The adaptation of freeform optics to a sub-wavelength metasurface platform allows for further miniaturization of optical components and offers a scalable route toward implementing near-arbitrary geometric curvatures in nanophotonics.

## Introduction

The function of an optical element is intrinsically tied to its geometry. While manufacturability has often constrained optical elements to have rotational invariance, the emerging field of freeform optics leverages more complex curvatures to enable novel functionalities and simplified compound optical systems^1–4^. These elements have been shown to be capable of correcting aberrations^3^, off-axis imaging^4^, expanding field of view^5^, and increasing depth of field^6^. Recent interest in freeform optics has been driven by potential applications in near-eye displays^7, 8^ as well as compact optical systems for medical, aerospace, and mobile devices where there are stringent constraints on the size and weight of the optical package^9^. One surface of particular interest is the cubic profile, where the surface of the optical element is defined by a cubic function. These elements have been shown to exhibit increased depth of focus^10, 11^, and in tandem, they can form an aberration-correcting lens with adjustable focus called the Alvarez lens^12, 13^. Many methods of realizing freeform optical elements, and in particular cubic surfaces, have been suggested and implemented, including fluid-filled^14^, custom single-point diamond turned polymer^15^, and diffractive optical elements^16^. Unfortunately, diffractive optics and single-point diamond turned elements often require complex and expensive fabrication, such as greyscale or multistage lithography or machining procedures ill-suited for large-scale production. Additionally, the thickness and working distances of these optical elements are large, resulting in an increased overall volume. Unlike conventional optics, metasurface optics design is curvature agnostic, readily accepting both conventional spherical curvatures as well as complex freeform surfaces with no additional design difficulties, while maintaining a thickness of the order of the optical wavelength. Moreover, well-developed semiconductor nanofabrication technology can be readily employed to fabricate such structures.

Metasurfaces are two-dimensional arrays of sub-wavelength scale scatterers arranged to arbitrarily control the wavefront of incident electromagnetic waves^17–19^. Rather than relying upon gradual phase accumulation, metasurfaces impart an abrupt, spatially varying phase profile on the incident light. This allows us to map complex curvatures onto a flat, wavelength scale thick surface by converting them into a discretized spatial phase profile. In addition to their compact size and weight, metasurfaces are fabricated using a single step lithography procedure with mature, highly scalable nanofabrication technology developed by the semiconductor industry. Numerous different metasurface material platforms have been demonstrated, including noble metals^17, 18, 20^, high contrast dielectrics^21–23^, and lower contrast dielectrics^24, 25^. For visible wavelengths, lower index dielectrics, such as silicon nitride, are desirable as they do not suffer from absorption losses due to their wide band gap, while exhibiting similar performance to other material platforms. In recent years, all dielectric metasurfaces have been used to build many different optical components such as quadratic lenses, vortex beam generators, and holograms^17, 18, 20, 21^. However, there has been little research in realizing imaging freefrom optical elements utilizing a metasurface platform. In this paper, we present a silicon nitride metasurface-based cubic phase optical element and an Alvarez lens operating at visible wavelengths. We observed an extended depth of focus (~300 μm), enough to ensure identical point spread function (PSF) for red and green light at the same image plane, potentially enabling white light imaging. Additionally, we experimentally demonstrated a change in focal length of ~2.5 mm by a physical displacement of only 100 μm using the Alvarez lens. The change in the focal length is significantly larger (~25 times) than the actual physical displacement, and is achieved by displacement perpendicular to the optical axis.

## Results

**Figure 1:**
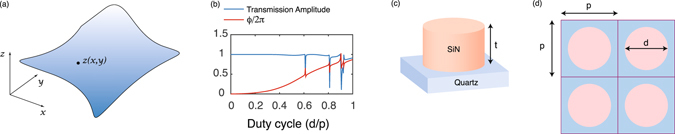
Mapping a freeform surface onto a metasurface: An arbitrary freeform surface is shown in (**a**). The corresponding height z(x, y) is converted into a discretized phase profile using the pillar parameters shown in (**b**). The parameters in (**b**) are capable of producing a full cycle of phase shifts and also maintain large regions of continuous, near unity transmission amplitude. (**c**) and (**d**) are simple schematics of a metasurface with thickness t, periodicity p, and diameter d.

In our metasurface design process, we take the sag profile of an arbitrary freeform surface, described by its height (*z*) as a function of its in-plane coordinates (*x*, *y*) as in Fig. 1a, and convert it into a discrete phase profile. We then quantize the phase profile into six linear steps from 0 to 2π corresponding to cylindrical posts with diameters *d* ranging from 192 nm to 420 nm using the corresponding values shown in Fig. 1b. We choose a set of parameters for posts with thickness *t* = λ, in this case 633 nm, arranged on a square lattice with periodicity *p* = *0.7 λ*, or 443 nm, (Fig. 1c,d).

Cubic phase elements have been explored for wave-front coding as part of a focus-invariant imaging system^10, 11^. These cubic phase elements do not cause incident light to converge into single point; incident rays converge along an extended length of the optical axis, allowing the point spread function (PSF) of the element to remain relatively constant for a large range of displacements along the optical axis. The resultant images from the optical system are often unintelligible to the human eye, but they can be digitally processed using knowledge of the cubic element’s PSF to recreate an image with enhanced depth of focus. More detail on the deconvolution process for the image is provided in the supplement S7. We design a cubic element with the phase profile:

1 $${\phi }(x,y)=mod(\frac{\alpha }{{L}^{3}}({x}^{3}+{y}^{3}),2\pi ),$$

where (x, y) are the device’s in plane coordinates, *L* is the width of the design, and *α* is a constant determining the rate of the phase variation on the metasurface. Larger values of *α* lead to better depth invariance at the expense of increased noise in the image while small values compromise the depth invariance^26^. Motivated by previous designs, we choose a value of α = 14π^26^.

The Alvarez lens is a compound optical element consisting of two cubic phase plates with one obeying the phase profile:

2 $${\phi }_{alv}(x,y)=mod(\frac{2\pi }{\lambda }A(\frac{1}{3}{x}^{3}+x{y}^{2}),2\pi )$$

and the other obeying its inverse such that *φ*_*alv*_(*x, y*) + *φ*_*inv*_(*x, y*) = 0, where (x,y) denotes the device’s in plane coordinate, and *A* is a constant determining the rate of phase variation on the metasurface. If the two elements are perfectly aligned, the Alvarez lens does not focus light, which can be interpreted as infinite focal length. Laterally displacing the elements relative to each other along the x-axis allows us to focus at finite lengths. Moreover, by controlling the extent of the lateral displacement along the x-axis we can change the focal length. Larger values of *A* increase the range of tunable focal lengths at the expense of image quality^13^. The range focal length with respect to displacements is given by the expression^12, 13^:

3 $$f=\,\frac{1}{4Ad},$$

where *f* is the focal length, *A* is the same constant as in the phase profile and *2d* is the relative displacement of the two surfaces meaning the Alvarez lens is displaced by a distance *d* and the inverse lens is displaced by *−d* from the origin. A derivation of the focal length expression is provided in the supplementary material S2. We emphasize that, unlike changing periodicity by stretching a metasurface lens^27^, this method can provide a much larger change in the focal length.

### Cubic metasurface

**Figure 2:**
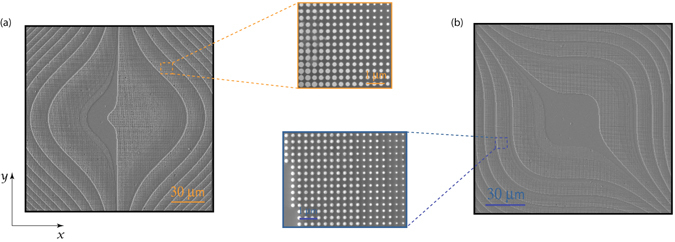
Scanning electron micrographs of fabricated devices coated in gold. Half of the Alvarez lens is shown in (**a**), and the cubic phase plate is shown in (**b**). Insets are zooms of specific locations of the metasurface showing the gradient in pillar sizes.

We fabricated a cubic metasurface with *α* = 14*π* and L = 150 μm, and a set of square Alvarez metasurfaces with *A* = 1.17 × 10^7^ m^−2^, and length 150 μm. The devices are fabricated in 633 nm silicon nitride deposited on top of a 500 μm fused quartz substrate. Scanning electron micrographs (SEMs) of the finished devices coated in gold are shown in Fig. 2.

**Figure 3:**
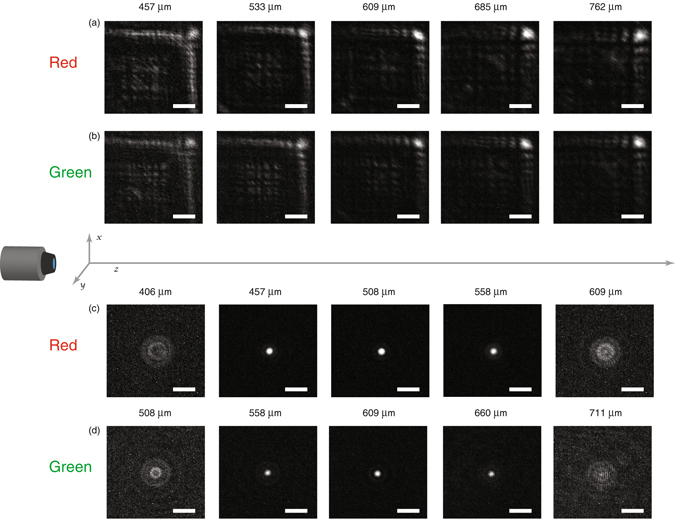
Dependence of cubic metasurface and metasurface lens point spread functions (PSF) upon displacement along the optical axis. (**a**) and (**b**) are the PSFs of the cubic element under coherent illumination by red and green light respectively. (**c**) and (**d**) are the PSFs of a 500 μm metasurface lens from ref. 24 under red and green illumination. All figures share the same 18 μm scale bar. The differences in the intensities of the images are due to the difference in incident intensities of the red and green lasers upon exiting the pinhole.

The cubic metasurface is characterized using a microscope free to translate along the optical axis. The device is mounted on a glass slide with the metasurface facing the microscope. Coherent illumination is provided using a red and a green laser sent through a pinhole. Note that the phase plates are designed to function with incoherent illumination^10, 11^, but the power of our LEDs was not high enough to determine the PSF. Intensity profiles were captured using the microscope and a CCD camera mounted on a translation stage. By translating the microscope and camera along the optical axis, we can image the x-y plane intensity profiles at varying z distances. To measure the PSF, we send each laser through the 5 μm diameter fixed pinhole. We use the microscope to image the PSF for varying displacements along the optical axis in order to characterize its afocal behavior. The measured PSFs are shown in Fig. 3 with respect to the z displacement. We see that indeed, the PSF of the element changes slightly with displacements along the optical axis of over 300 μm, confirming the depth-invariant behavior of the cubic phase plate. In addition to the measurement of the PSF, we also calculated and compared the modulation transfer function (MTF), shown in supplement S7. The calculated MTFs are very similar for the cubic metasurfaces. For comparison, we also measured the PSF of a metasurface lens (quadratic phase profile) with a focal length of 500 μm shown in Fig. 3c,d. It is clear that the PSF of the lens is highly dependent upon displacements along the optical axis, changing completely over a range of 100 μm, unlike that of the cubic phase plate. While the cubic metasurface exhibited a large range of displacements for which the PSFs were similar for the two illumination wavelengths (red and green), the metasurface lenses show significant chromatic aberrations. With the understanding that an image is the convolution of an object with the imaging system’s impulse response or PSF, this effect could be used to perform white light imaging. If the PSF is identical for a range of wavelengths, deconvolution of the image can be performed with a single digital filter obtained from the imaging system’s PSF^28^. For highly chromatic optical elements, this is not possible as shown in Fig. 3c,d, but we can utilize the cubic element’s increased depth of focus to find a point where the PSF is the same for a range of wavelengths. We note that this may truly enable broadband operation, unlike previously reported results, where the lens only works for certain discrete wavelengths^29, 30^.

### Alvarez lens

**Figure 4:**
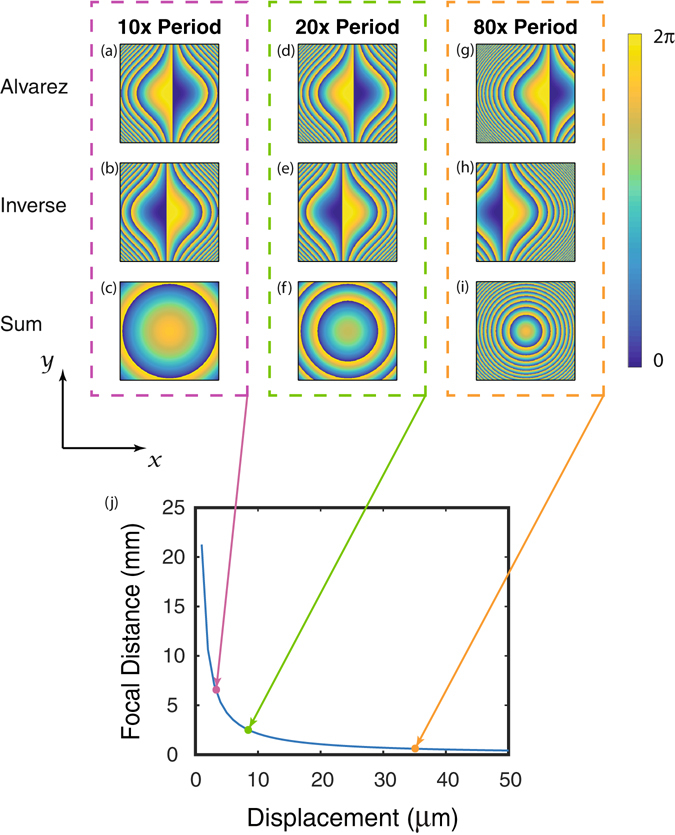
Behavior of the Alvarez lens in response to x displacement. (**a**,**d**,**g**) represent the phase profiles of one Alvarez element for displacements of 10p, 20p, 80p respectively, (**b**,**e**,**h**) represent their inverses at displacements of −10p, −20p, −80p respectively, and (**c**,**f**,**i**) are the sums of the displaced phase profiles. The phase profiles are displaced in units of the metasurface lattice periodicity p = 443 nm, with (**a**–**c**) representing a 4.43 μm displacement, (**d**–**f**) representing 8.86 μm displacement, and (**g**–**i**) representing a 35.4 μm displacement. (**j**) Plot of focal length dependence on displacement based on equation 4. Larger displacements result in a more rapidly varying phase profile, corresponding to a lens with a smaller focal length. The colored dots indicate the focal lengths of lenses shown in (**c**,**f**,**i**). Parameters used are the same as for the fabricated device, L = 150 μm, A = 1.17 × 10^7^m^−2^.

To characterize the Alvarez lens, we mount each metasurface on its own glass slide with the metasurfaces facing each other to minimize the axial separation between them. Simulation and experimental data on axial separation is presented in supplement S6. The metasurface close to the objective is mounted on a thinner glass slide in order to allow us to image short focal lengths, and then placed on a fine translation stage with steps as fine as 0.5 μm for displacements along the x direction. Illustrations of the behavior of the Alvarez phase profile for displacements along the x axis are shown in Fig. 4. For small displacements, the resulting phase profile is slowly spatially varying, corresponding to a lens with a large focal length, while large displacements correspond to a highly varying phase profile, or a short focal length lens. The theoretical performance of the lens based on the previous formula for our design parameters is shown in Fig. 4j.

**Figure 5:**
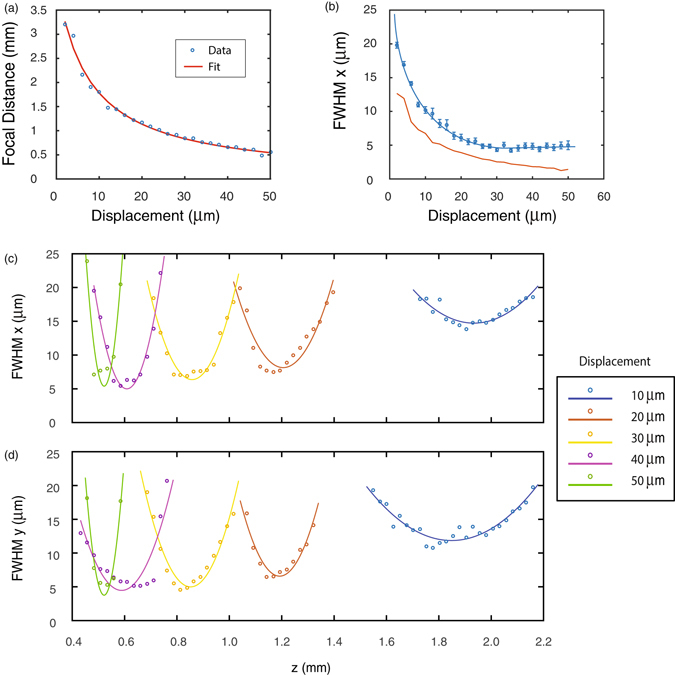
Alvarez lens performance. (**a**) Measured focal distance of the Alvarez lens pair plotted against x displacement. The red line is a theoretical fit to the focal length data. (**b**) Full width at half maximum (FWHM) measured along the x axis plotted against x displacement. The measured data are shown as blue points while the blue line is an eye guide. The diffraction-limited spot size FWHM is plotted in red. Error bars represent a 95% confidence interval of a Gaussian fit. For both (**a**) and (**b**) images were taken with a displacement step size of 2 μm. (**c**,**d**) Behavior of the Alvarez lens FWHM for five displacements along x-axis. The FWHM of the spot-size in the sensor plane is plotted as the microscope moves into and out of the focal plane. The FWHMs are measured along the (**c**) x and (**d**) y axes. FWHM data is plotted as the points, and the lines are eye guides.

We have experimentally measured the focal lengths for displacements *d* of each metasurface from 2 to 50 μm and find the focal distances change from a minimum of 0.5 mm to a maximum of 3 mm as seen in Fig. 5a. This indicates that with a physical displacement of 100 μm, the focal length changes by 2.5 mm corresponding to a change in optical power (inverse focal length) of about 1600 diopters. While the experimentally measured change in the focal length is significantly smaller than the simple theoretical predictions in Fig. 4i, this change is still a significant change in focal length by a mechanically actuated metasurface-based tunable optical element^27, 31, 32^. In addition, we emphasize that the lens achieves most of its focal tuning range at a small range of physical displacement, in that we can tune the focal length by 2 mm using only around 30 μm of physical displacement. We performed a simple fit of the form:

4 $$f({\rm{d}})=\,\frac{1}{4{\rm{A}}({\rm{d}}+{\rm{B}})},$$

to generate the red line shown in Fig. 5a. The best fitting parameters are A = 7.97 × 10^6^ m^−2^, similar to our design value of 1.17 × 10^7 ^ m^−2^ and B = 7.6 μm, which indicates the extent of misalignment. We believe the major sources of the discrepancy between the measurement and the theoretical prediction are this small degree of misalignment (of order B) and also the discretized phase profile of the metasurface, in contrast to the continuous profile assumed in the theory. The effect of discretized phase is verified via FDTD simulations of a metasurface-based Alvarez lens presented in the supplementary material S1. Previous focus-tunable metasurface lenses were based on stretchable substrates, which have a focal length dependence *f* ∝ (1 + *∈*)^2^ in the paraxial limit where *∈* is the stretching factor^27^, corresponding to a change Δ*f* = (2*∈* + *∈* ^2^)*f*. This change is linear to first order in *∈* with the quadratic term dominating for greater than unity stretch factors, whereas the change in focal length of the Alvarez lens behaves nonlinearly as shown in the equation (2) (details provided in the supplementary material S2), with the changes in focal length occurring for the smallest physical displacements. Another important quantity to assess the quality of a lens is the spot size, which we measure by calculating the full width at half maximum (FWHM) of a Gaussian fit to a 1D slice of the intensity data. The FWHM shows a similar dependence on lateral displacement as the focal length. The largest focal length of ~3 mm displays the largest FWHM of ~20 μm, while the smallest focal length of ~0.5 mm has a FWHM of ~5 μm (Fig. 5a). We find that our measured FWHM is around twice that of the diffraction-limited spot size using the methodology in ref. 22 (Fig. 5b). In addition, we characterize the behavior of the lens as it moves into and out of the focal plane as shown in Fig. 5c,d. The FWHM of the lens is measured using a horizontal (x) and vertical (y) 1D cross-sections for Fig. 5c,d, respectively. Mirroring the results from our numerical simulations (supplementary materials S1, S4), the beam spot is wider along the x than along the y axis.

## Discussion

We have fabricated and demonstrated the performance of a metasurface-based cubic phase element and Alvarez lens in silicon nitride. To the best of our knowledge, this is the first metasurface-based Alvarez lens. We believe this metasurface platform is near ideal for both adapting existing freeform optical elements, and also realizing new classes of arbitrary spatial phase profiles. This platform also has the unprecedented ability for the integration of freeform optical elements at the micron scale leading to ultra-miniature optical systems. For example, throughout tunable optical designs, we find that a mechanical change of *x* nm results in a change in focal length or in resonance wavelength of the order *O*(*x*) nm^33, 34^. In the case of the Alvarez lens no such limitations exist, and we demonstrated greater than 2 mm focal length tuning, with only tens of microns physical displacement. Such a small displacement is beneficial, especially if the displacement is realized using integrated MEMS devices. Similarly, by using a non-quadratic phase profile, we can potentially realize white light imaging in diffractive optics. In particular, our results indicate a depth-invariant point spread function for red and green lasers for the cubic phase-mask, resulting in the same PSF for both colors at the image plane. The reported metasurfaces involving cubic phase profiles represent a first step towards the promising new field of metasurface-enabled freeform optics, which will find applications in correcting aberrations, building compact optical systems or sensors, such as realizing near-eye displays^7, 8^ or ultra-compact endoscopes^9^. Additionally, by adapting existing semiconductor technologies, such as nano-imprint lithography, these devices can easily be fabricated in a scalable manner.

## Methods

### Simulation and design

To arrive at the parameters shown in Fig. 1b, we calculate the scattering properties of different pillar configurations using rigorous coupled-wave analysis (RCWA) in order to obtain 0 to 2π phase shifts while maintaining near unity amplitude transmission coefficients. The calculation was done for wavelength λ = 633 nm for silicon nitride pillars with index of refraction n = 2, thickness t = λ, periodicity p = 0.7 λ, and diameter d varying from 0 to the period p (duty cycle varying from 0 to 1). The simulation was performed assuming a substrate of fused silica (n = 1.5).

The Alvarez phase-plate obeys a sag profile:

5 $$z(x,y)=A(\frac{1}{3}{x}^{3}+x{y}^{2}),$$

which is converted into a phase profile for operation at λ = 633 nm by the multiplication of the corresponding free space wave vector, giving us equation (2). The resulting phase profile is discretized in x-y plane on a square grid of periodicity p = 0.7 λ and then quantized in six linear steps between 0 and 2π using the geometric parameters shown in Fig. 1b. In order to test the compatibility of the Alvarez lens with adaptation to a metasurface, we performed finite-difference time-domain (FDTD) simulations with a design of *A* = 6.67 × 10^9^ m^−2^. The focal length and FWHM behavior is shown in supplement S1. We observed focusing between 8 μm and 40 μm for this lens. The actual lens design presented in this manuscript was not tested using FDTD simulations due to computational resource restrictions.

### Sample Fabrication

Both the devices shown in Fig. 2 were fabricated in a 633 nm thick silicon nitride (SiNx) deposited on a 0.5 mm fused quartz substrate. We used plasma-enhanced chemical vapor deposition (PECVD) techniques to deposit the silicon nitride at a temperature of 350 °C. Following PECVD, we evaporated  50nm of aluminum to serve both as a charge-dissipation layer for electron beam lithography (EBL) and also as a hard mask. Electron beam resist (140 nm, ZEP 520 A 1:1 in anisole) was then spin-coated on top of the wafer. We wrote the pattern using a JEOL JBX-6300FS 100 kV EBL system. After development in amyl acetate, the sample was dry-etched using an Cl_2_ and BC1_3_ chemistry to transfer the pattern on the aluminum layer, forming a hard mask. Finally, a CHF_3_ and O_2_ chemistry was used to dry-etch 633 nm pillars, and the remaining aluminum was removed using AD-10 photoresist developer. For capturing the scanning electron micrographs in Fig. 2, the samples were coated in gold as a charging layer as both silicon nitride and the fused quartz substrate are insulators.

### Measurement Procedure

The cubic phase plate measurements were performed on a setup shown in supplementary Fig. S2. Light coupled from a helium-neon laser was coupled to a fiber for the red measurements, and light from a 532 nm laser was used for green measurements. The light was sent through a 5 μm fixed pinhole (Thorlabs P5S) before illuminating the sample mounted on a standard 1 mm glass microscope slide with the metasurface facing the microscope. The cubic PSFs were measured using 4 mW of incident power onto the pinhole, and the lenses PSFs were measured using 1.5 mW of incident power. A home-built microscope comprising of a 40x objective (Nikon Plan Fluor) with a working distance of 0.66 mm and NA 0.75 and a tube lens (Thorlabs ITL200) with a focal length of 20 cm is used to measure the field profiles. This microscope images the intensity profile generated by the cubic phase plate onto a Point Grey Chameleon CCD. The magnification of the setup was determined using known dimensions of the fabricated metasurface. By translating the microscope along the optical axis (z) we were able to image the intensity profile in steps of 25.4 μm to capture the images shown in Fig. 2.

The Alvarez lens performance was measured using a setup shown in supplementary Fig. S3. Red light is obtained from a fiber-coupled light-emitting diode (Thorlabs M625F1) and directed towards the sample. The Alvarez lens consists of the Alvarez phase plate and the inverse phase plate, and the two samples are mounted with the devices facing each other. The Alvarez phase plate is mounted on a standard 1 mm glass slide while the inverse phase plate is mounted on a thin glass coverslip with a thickness between 0.16 to 0.19 mm (Fisherbrand 12-544-E). The Alvarez phase plate is placed on the illumination side while the inverse phase plate is placed on the microscope side. Finally, the Alvarez phase plates were mounted on an x-z translation stage enabling control over the displacement between the two phase plates in the x and z directions. The x direction can move in increments as fine as 0.5 μm.

The focal distance of the Alvarez lens is measured for displacements of 2 μm to 50 μm in steps of 2 μm. For each displacement, the microscope is translated along the z axis, imaging intensity profiles in steps of 25.4 μm. Due to the sensitivity of the focal length to small misalignments, all data was taken consecutively from one displacement to the next with one alignment at the beginning of the measurement. Measurements for five displacement values showing the microscope moving into and out of the focal plane are shown in Fig. 5c,d. The alignment of the two metasurfaces is done one at a time by first imaging the first metasurface on the CCD and marking a single corner with a marker. The microscope is then translated backwards along the optical axis to allow us to bring the second metasurface into focus and translate its corner to the same marker. Finally, the two metasurfaces are translated along the optical axis to minimize their separation by eye. In order to minimize the separation between the two elements, both Alvarez lenses were mounted on stages free to move along the optical axis. The distance between the two was determined using the microscope by focusing on each element separately and recording their positions. The two elements were then brought together to their final separation of less than 0.3 mm. We did not bring the elements closer because of the possibility of scratching the elements.

The FWHM of the focal spot for each x displacement is determined by taking the minimum FWHM from a Gaussian fit to a 1-D slice of the intensity data for each z displacement as described in the supplementary material S4. The focal distance is then determined by taking the z displacement value of the minimum FWHM. The FWHM is measured for both the x and y directions. The diffraction-limited spot FWHM is obtained from a Gaussian fit to a lens with the same diameter and the measured focal length for that displacement.

## Electronic supplementary material

Supplementary Material

## References

[1] Kaya I, Thompson KP, Rolland JP (2012). Comparative assessment of freeform polynomials as optical surface descriptions. Opt. Express.

[2] Thompson KP, Rolland JP (2012). Freeform Optical Surfaces: A Revolution in Imaging Optical Design. Opt. Photon. News.

[3] Fuerschbach K, Rolland JP, Thompson KP (2014). Theory of aberration fields for general optical systems with freeform surfaces. Optics Express.

[4] Fuerschbach K, Davis GE, Thompson KP, Rolland JP (2014). Assembly of a freeform off-axis optical system employing three;-polynomial Zernike mirrors. Optics Letters.

[5] Nie Y, Duerr F, Thienpont H (2015). Direct design approach to calculate a two-surface lens with an entrance pupil for application in wide field-of-view imaging. OPTICE.

[6] Duerr F, Meuret Y, Thienpont H (2013). Potential benefits of free-form optics in on-axis imaging applications with high aspect ratio. Optics Express.

[7] Cakmakci, O. *et al*. In *Mixed and Augmented Reality, ISMAR 2008. 7th IEEE/ACM International Symposium on*. 29–32 (2008).

[8] Hua, H. Sunglass-like displays become a reality with free-form optical technology. *SPIE Newsroom* (2012).

[9] Rege SS, Tkaczyk TS, Descour MR (2004). Application of the Alvarez-Humphrey concept to the design of a miniaturized scanning microscope. Optics Express.

[10] Dowski ER, Cathey WT (1995). Extended depth of field through wave-front coding. Appl. Opt..

[11] Bradburn S, Cathey WT, Dowski ER (1997). Realizations of focus invariance in optical–digital systems with wave-front coding. Appl. Opt..

[12] Alvarez, L. W. inventors; Optical Res & Dev Corp., assignee; Two-element variable-power spherical lens. US patent, US 3305294 A (Feb 21, 1967).

[13] Barbero S (2009). The Alvarez and Lohmann refractive lenses revisited. Optics Express.

[14] Li L (2014). Fabrication of microinjection-molded miniature freeform Alvarez lenses. Appl. Opt..

[15] Zhou G, Yu H, Chau FS (2013). Microelectromechanically-driven miniature adaptive Alvarez lens. Optics Express.

[16] Barton IM (2000). Diffractive Alvarez lens. Optics Letters.

[17] Kildishev AV, Boltasseva A, Shalaev VM (2013). Planar Photonics with Metasurfaces. Science.

[18] Yu N, Capasso F (2014). Flat optics with designer metasurfaces. Nat Mater.

[19] Yu N (2011). Light Propagation with Phase Discontinuities: Generalized Laws of Reflection and Refraction. Science.

[20] Aieta F (2012). Aberration-Free Ultrathin Flat Lenses and Axicons at Telecom Wavelengths Based on Plasmonic Metasurfaces. Nano Letters.

[21] Arbabi, A., Horie, Y., Bagheri, M. & Faraon, A. Dielectric metasurfaces for complete control of phase and polarization with subwavelength spatial resolution and high transmission. *Nat Nano* advance online publication, doi:10.1038/nnano.2015.186 (2015). 10.1038/nnano.2015.186 26322944

[22] Arbabi, A., Horie, Y., Ball, A. J., Bagheri, M. & Faraon, A. Subwavelength-thick lenses with high numerical apertures and large efficiency based on high-contrast transmitarrays. *Nat Commun* **6**, doi:10.1038/ncomms8069 (2015). 10.1038/ncomms8069 25947118

[23] Khorasaninejad M (2016). Metalenses at visible wavelengths: Diffraction-limited focusing and subwavelength resolution imaging. Science.

[24] Zhan A (2016). Low-Contrast Dielectric Metasurface Optics. ACS Photonics.

[25] Astilean S, Lalanne P, Chavel P, Cambril E, Launois H (1998). High-efficiency subwavelength diffractive element patterned in a high-refractive-index material for 633nm. Optics Letters.

[26] Mirotznik MS, van der Gracht J, Pustai D, Mathews S (2008). Design of cubic-phase optical elements using subwavelength microstructures. Opt. Express.

[27] Ee H-S, Agarwal RT (2016). Metasurface and Flat Optical Zoom Lens on a Stretchable Substrate. Nano Letters.

[28] Wach HB, Dowski ER, Cathey WT (1998). Control of chromatic focal shift through wave-front coding. Appl. Opt..

[29] Aieta F, Kats MA, Genevet P, Capasso F (2015). Multiwavelength achromatic metasurfaces by dispersive phase compensation. Science.

[30] Arbabi, E., Arbabi, A., Kamali, S. M., Horie, Y. & Faraon, A. Multiwavelength polarization insensitive lenses based on dielectric metasurfaces with meta-molecules. *arXiv:1601.05847* (2016).

[31] Zhu L, Kapraun J, Ferrara J, Chang-Hasnain CJ (2015). Flexible photonic metastructures for tunable coloration. Optica.

[32] Gutruf P (2016). Mechanically Tunable Dielectric Resonator Metasurfaces at Visible Frequencies. ACS Nano.

[33] Yu CL (2012). Stretchable Photonic Crystal Cavity with Wide Frequency Tunability. Nano Letters.

[34] Zeng B, Majumdar A, Wang F (2015). Tunable dark modes in one-dimensional “diatomic” dielectric gratings. Optics Express.

