# Supplementary material for: Metasurface Freeform Nanophotonics
Source: Sci Rep. 2017 May 10;7:1673. doi: 10.1038/s41598-017-01908-9 (PMC5431933; doi:10.1038/s41598-017-01908-9)
Supplement: Supplementary file 1 — Supplementary Material [file 41598_2017_1908_MOESM1_ESM.pdf]

Supplement: Metasurface Freeform Nanophotonics

Alan Zhan [1], Shane Colburn [2], Christopher M. Dodson [2], Arka Majumdar [1,2]

[1]Department of Physics, University of Washington, Seattle, WA-98195, USA

[2]Department of Electrical Engineering, University of Washington, Seattle, WA-98195, USA

S1. Simulation results

S2. Alvarez Focal Length Formula Derivation

S3. Setups

S4. Measurement and Diffraction Limit

S5. Alvarez Chromatic Behavior

S6. Alvarez Axial Separation Behavior

S7. Cubic Image Retrieval

S1: Simulation Results

We performed finite-difference time-domain (FDTD) simulations of the metasurface-based Alvarez lens to understand the effect of discretization of the phase profile. We find the change in the focal length qualitatively matches the theoretical predictions assuming a continuous phase profile, but the numerically calculated focal lengths do not quantitatively match well with the theoretical equation derived for a continuous phase profile. In particular, the focal lengths deviate significantly at small displacement, as we also observed in our experiment. Additionally, we find the focal spot size is larger in the x direction than in the y direction, also in accordance with experiment. In calculating the diffraction limit for the x direction, we account for an increase in the physical lens size due to the displacement along that axis. This accounts for the differences in diffraction limits shown in Fig S1c for the x and y directions.

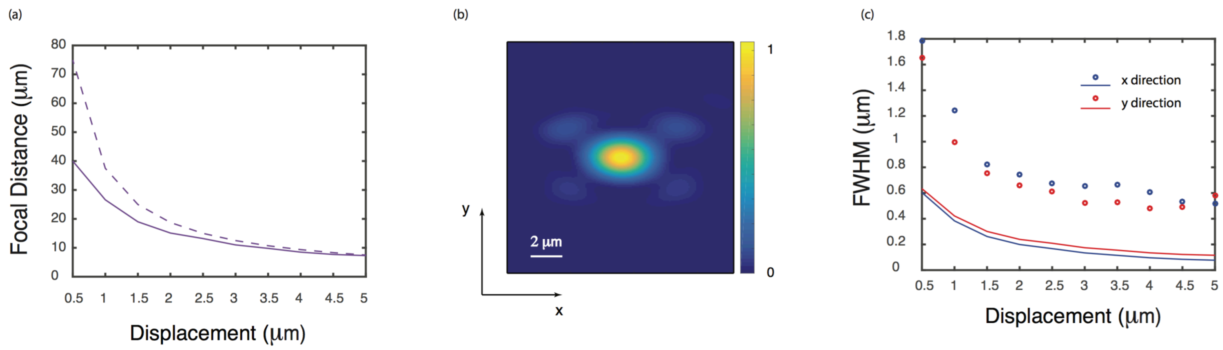

Fig. S1: FDTD simulation results for an Alvarez lens. (a) the measured focal length plotted against lateral displacement. The simulation data is shown as the solid line, and the theoretical focal length range (assuming a continuous phase profile) is shown as the dotted line. Displacements are made in steps of 0.5 µm. (b) an example of a simulated focal spot for a 0.5 µm displacement. (c) The numerically estimated FWHM for each displacement step of 0.5 µm. The x and y FWHM are plotted as points that are blue and red respectively. The calculated diffraction limit corresponding to the x and y geometric parameters of that lens are shown as solid lines in blue and red respectively. Parameters for simulation are A = 6.67×10^{9} m^{-2}, and the phase plates are 10 µm x 10 µm.

S2: Alvarez Focal Length Formula Derivation:

The central concept of the Alvarez lens is the dependence of the focal length on the lateral displacement of the two phase plates, the Alvarez phase plate obeying:

$$\varphi_{Alv}(x,y) = A\left(\frac{1}{3}x^3 + xy^2\right), (1)$$

and the inverse phase plate obeying its negative:

$$\varphi_{Inv}(x,y) = -A\left(\frac{1}{3}x^3 + xy^2\right), (2)$$

such that $\varphi_{Inv}(x,y) + \varphi_{Alv}(x,y) = 0$ for aligned phase plates. For a displacement d along the x axis, the addition of the two surfaces produces a quadratic phase profile plus a constant phase offset:

$$\varphi_{Sum}(d) = \varphi_{Alv}(x+d,y) + \varphi_{Inv}(x-d,y) = 2Ad(x^2+y^2) + \frac{2}{3}d^3, (3)$$

neglecting the constant phase offset, and setting $r^2 = (x^2 + y^2)$, we recognize the expression for a lens under the paraxial approximation:

$$\varphi_{Lens}(d) = 2Adr^2 = \frac{r^2}{2f}, (4)$$

with focal length as a function of displacement:

$$f(d) = \frac{1}{4Ad}, (5)$$

S3: Setups

The experimental setups are shown in Fig. S2 and S3.

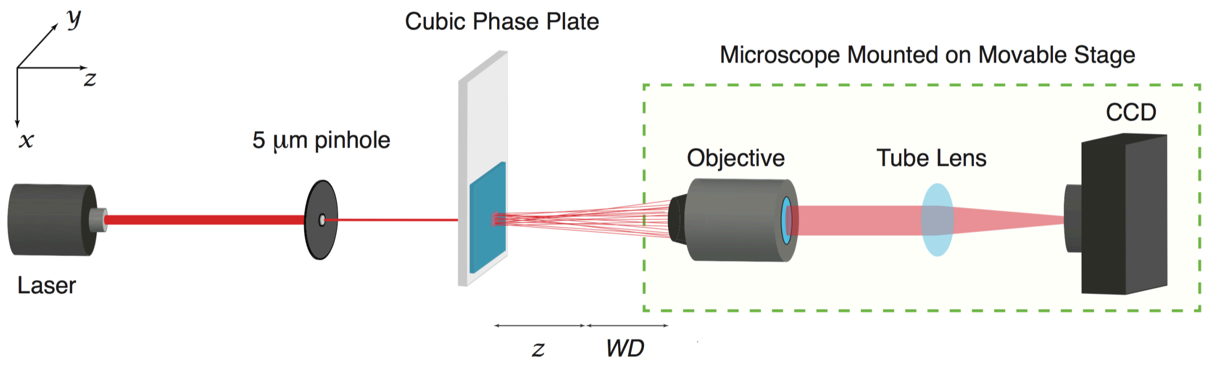

Fig. S2: Point spread function measurement setup: Schematic of the setup used to measure the point spread functions of the cubic metasurface phase plate and the metasurface lens. Illumination is provided either by a helium-neon laser for red or a 532 nm laser for green, and is passed through a 5 µm pinhole to approximate a point source. The microscope is free to move along the z axis.

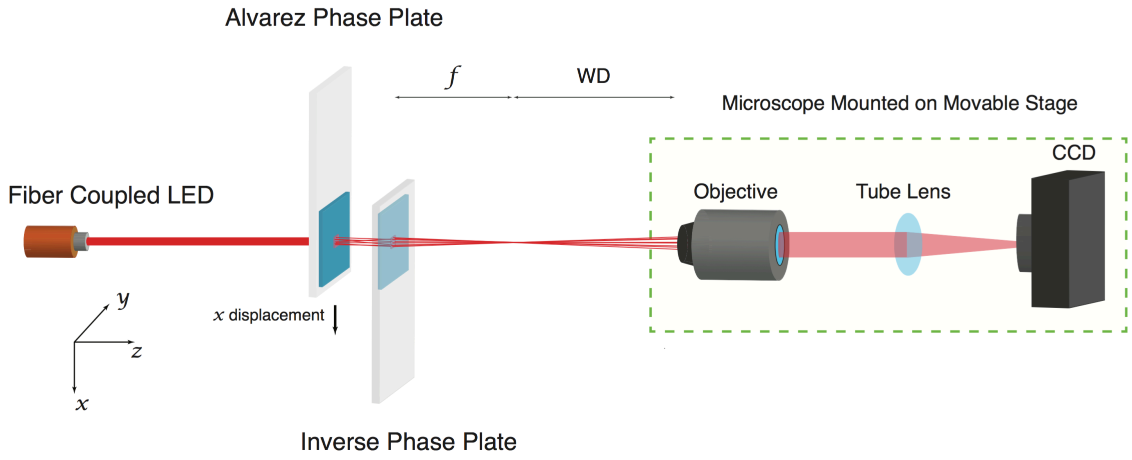

Fig. S3: Alvarez phase plate measurement setup: Schematic of the setup used to measure the performance of the Alvarez lens. Light is provided by a fiber-coupled red light-emitting diode (LED). The Alvarez phase plate is mounted on the LED side while the inverse phase plate is mounted on the microscope side. The Alvarez phase plate is allowed to move in the x direction. The microscope is free to move along the z axis, allowing us to image into and out of the focal plane for each displacement.

S4: Measurement and Diffraction Limit

The experimentally measured focal spot from the Alvarez lens shows different FWHM along x and y direction, which is consistent with the numerical FDTD simulations (Fig S1). Here we present our criterion for characterizing the focusing performances of a lens based on its FWHM. An ideal lens with focal length f and radius d will have an Airy disk intensity profile given by:

$$I(\theta) = I_o \left(\frac{2J_1(kd \sin\theta)}{kd \sin\theta} \right)^2$$

where I_o is the central peak intensity, $J_1(x)$ is the first order Bessel function of the first kind, k is the free space wave vector of the illuminating light, d is the lens radius, and θ is the angular position. The diffraction-limited FWHM for a particular lens with geometric parameters f and d is obtained by a Gaussian fit to the Airy disk intensity profile.

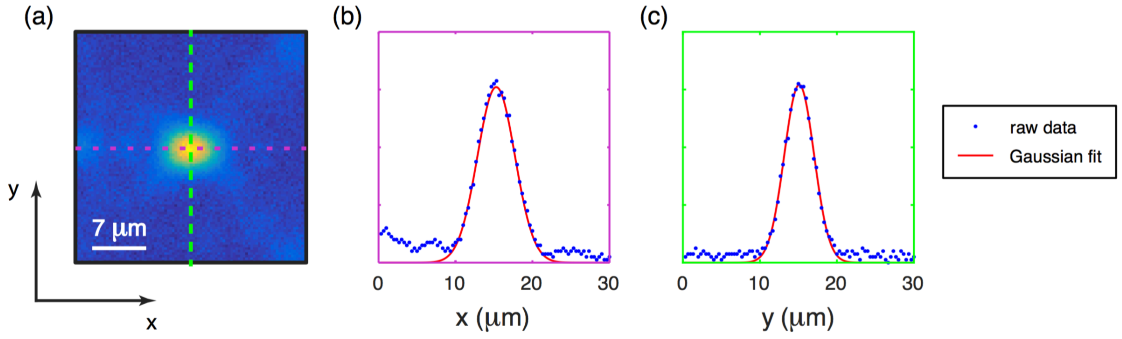

Fig. S4: Characterizing full width half maximum: (a) an example of an experimental focal spot for an Alvarez lens with 30 μm lateral displacement. A Gaussian fit is used along the x (b) and y (c) axes to estimate the focal spot size.

S5: Alvarez Chromatic Behavior

The same lens as in supplement S1 was simulated at a displacement of 4 μm for wavelengths between 400 to 700 nm in steps of 50 nm. The electric field intensities in the x-z and y-z planes centered at the optical axis are plotted in Fig S6 for the range of simulated wavelengths. We find that the Alvarez lens fails to focus adequately at wavelengths below 550 nm, and displays expected chromatic aberrations in the wavelength range 550 nm to 700 nm. At 400 and 450 nm, the wavelength of light is less than and approaching the periodicity respectively, so we do not expect the Alvarez lens to perform well in that regime.

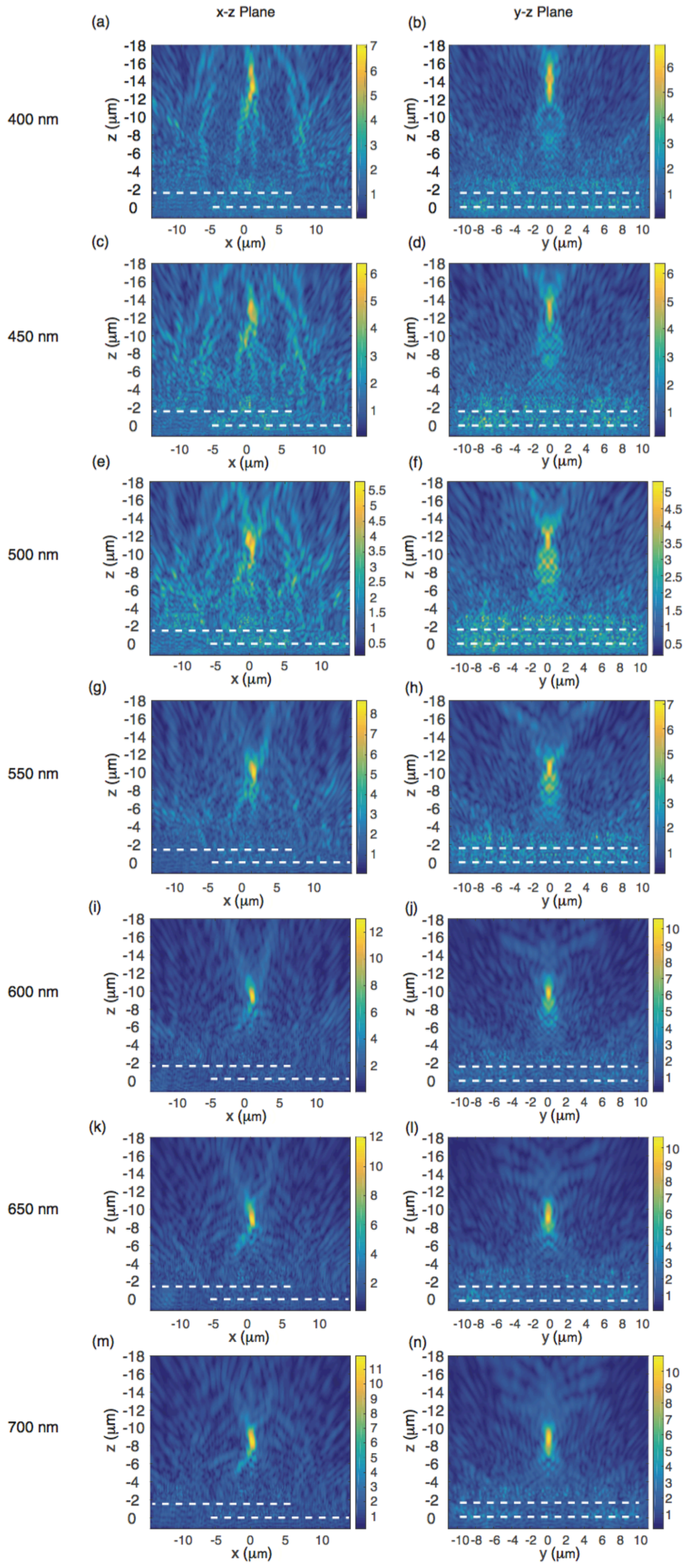

x-z Plane
y-z Plane
(a)
(b)
(c)
(d)
(e)
(f)
(g)
(h)
(i)
(j)
(k)
(l)
(m)
(n)
400 nm
450 nm
500 nm
550 nm
600 nm
650 nm
700 nm
z (µm)
x (µm)
y (µm)

Fig S5: Chromatic behavior of the Alvarez lens. Plotted are the electric field intensity profiles in the x-z and y-z planes centered at the optical axis for illumination wavelengths covering the visible spectrum in steps of 50 nm. The lens begins to form a distinct focal spot for 550 nm in both the x-z and y-z planes. The white dashed lines indicate the locations of the two metasurfaces comprising the Alvarez lens.

S6: Alvarez Axial Separation Behavior

We investigated the dependence of the Alvarez lens's focusing behavior both in FDTD simulation and also in experiment. The two Alvarez plates can be understood as generating Airy beams accelerating along a parabolic path on the axis of displacement (x for our design). As the axial separation between the metasurfaces becomes larger, the initial Airy beam generated by the first metasurface begins to diverge away from the second plate, which has finite extent, causing degradation of the focal spot.

In simulation, as shown in Fig. S6, axial displacements have a large effect on the shape of the focal spot in the x-z plane, and also a large effect on the intensities of the focal spots for both planes. As seen in Fig. S6 (d), for large separations, the Airy beam generated by the first metasurface begins to clip the edge of the second.

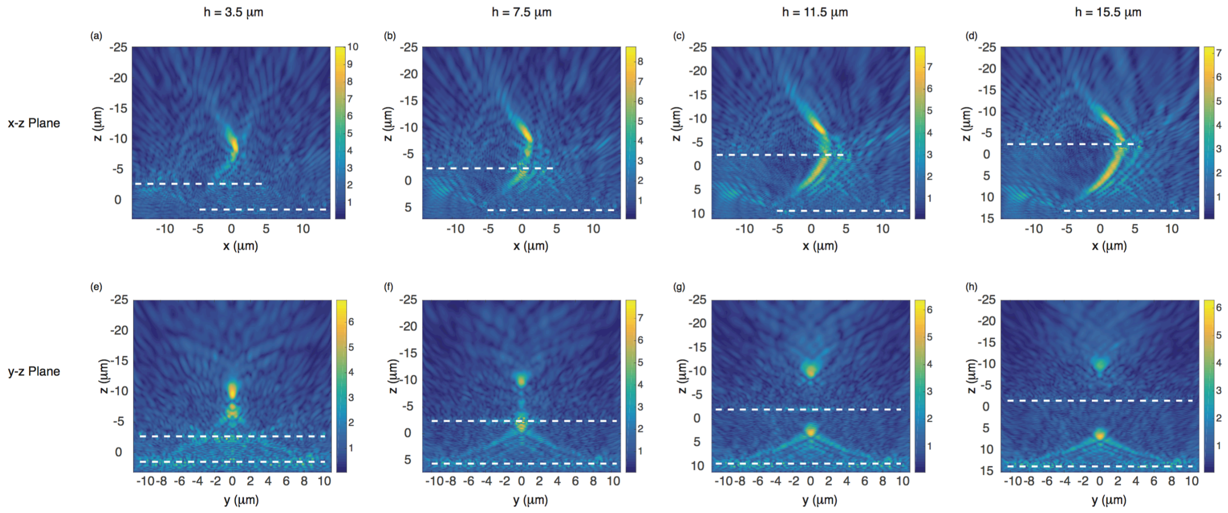

Fig. S6: Simulated Alvarez lens performance for displacements along the optical axis. Plotted are the electric field intensities. As the displacement increases, the x-z plane focal spot deforms, elongating, and also decreasing in intensity (a)-(d). However the focal spot remains near 10 μm. In the y-z plane, the focal spot remains near 10 μm and retains its shape, but decreases rapidly in intensity (e)-(h). The simulated design is the same as in supplement S1, and has an in plane displacement of 4 um. The axial displacement is represented by h, and the dashed white lines represent the locations of the two metasurfaces comprising the Alvarez lens.

In experiment, the axial displacement decreased the focal distance of the lens along both the x and y axes of the lens (Fig S7). However, the effect on the focal spot size was not deterministic, showing large spikes in one set of data and a gradual increase in the other. The metasurface near the objective remained stationary while the metasurface near the illumination source was translated backwards to increase the separation. From this result we see the separation does have an effect on the focal distance of the metasurface, but not enough to account for the large discrepancy between theoretical and experimental performance.

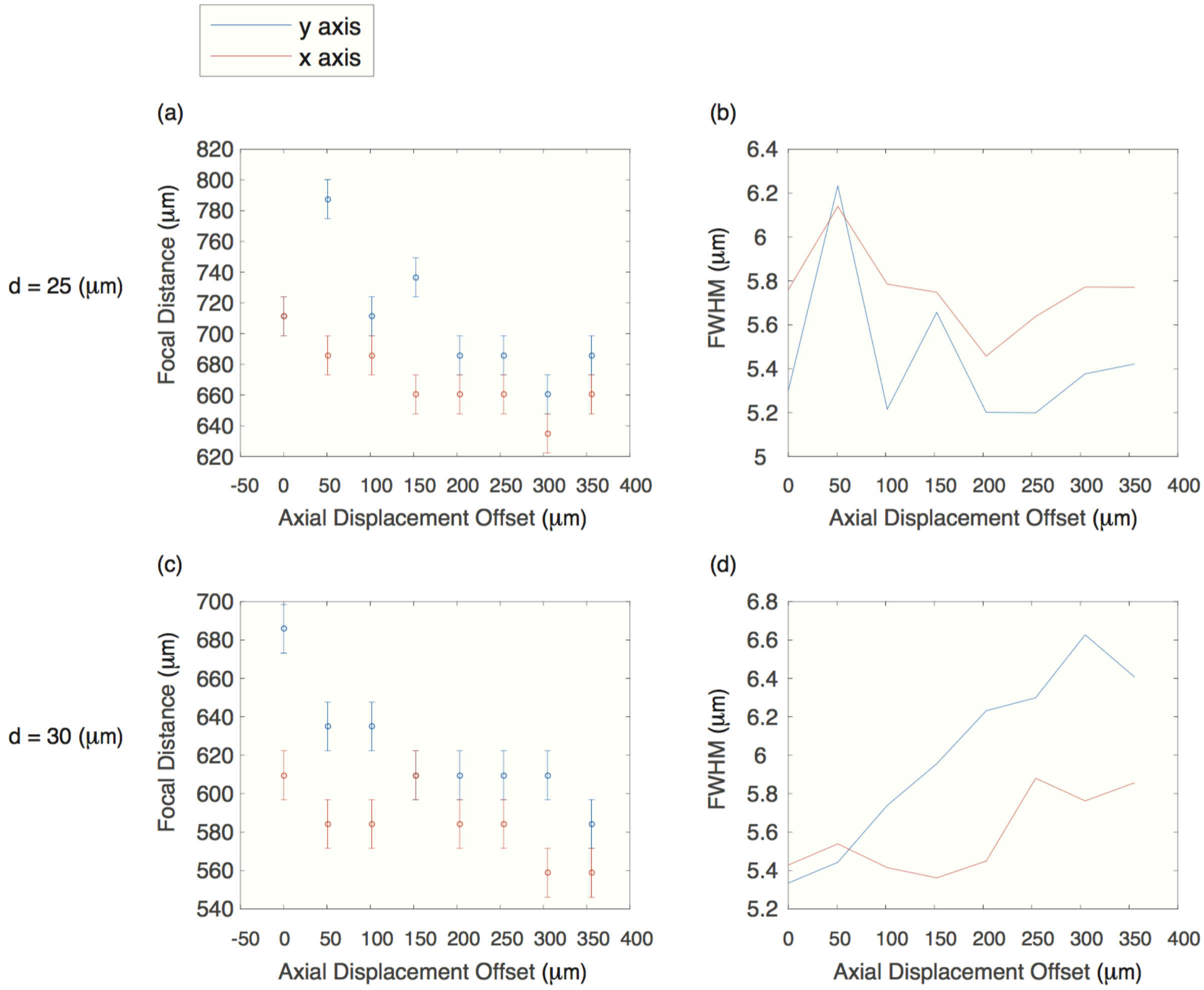

Fig. S7: Experimental Alvarez lens performance for displacements along the optical axis. (a), (c) focal distances for an Alvarez lens with 25 and 30 μm of transverse displacement d, respectively As the displacement increases, both displacements displayed a decrease in focal length. The axial displacement is not absolute, and can be thought of as an offset of some finite distance. (b), (d) show the effect of the displacement on the focal length of the Alvarez lens for 25 and 30 μm of axial displacement respectively. Data in red and blue represent data taken from the y and x axes respectively. Error bars represent the mechanical error associated with our translation stage.

S7: Cubic Image Retrieval

In order for the cubic imaging system to provide useful images, the initial image must be post processed by deconvolving the cubic point spread function (PSF) from the initial image[1]. In

order for the cubic phase plate to be useful in controlling chromatic aberrations, the PSF must be the same for the wavelengths of interest in some region of space. This is in general not possible for highly chromatic optical elements such as metasurfaces, but the metasurface cubic phase plate does satisfy this criteria for 633 nm and 532 nm illumination.

We quantify this invariance by calculating the modulation transfer function (MTF) of our experimentally measured PSFs using a two dimensional Fourier transform, shown in Fig S8 for the cubic elements and Fig S9 for the quadratic elements. The figures are 1D slices of a 2D MTF and we are justified in taking a 1D MTF due to the rectangular separability of the phase function[2]. As shown in Figs S8 and S9, the cubic phase plate under green and red illumination exhibits very similar MTFs for a range of positions along the optical axis while the quadratic lens fails. Notably for the cubic MTFs, the positions of the peaks and troughs are similar for low frequency components while this is not true for the quadratic elements. Using the knowledge of our experimental PSF and MTF, a frequency domain filter can be constructed using a least squares optimization routine[3].

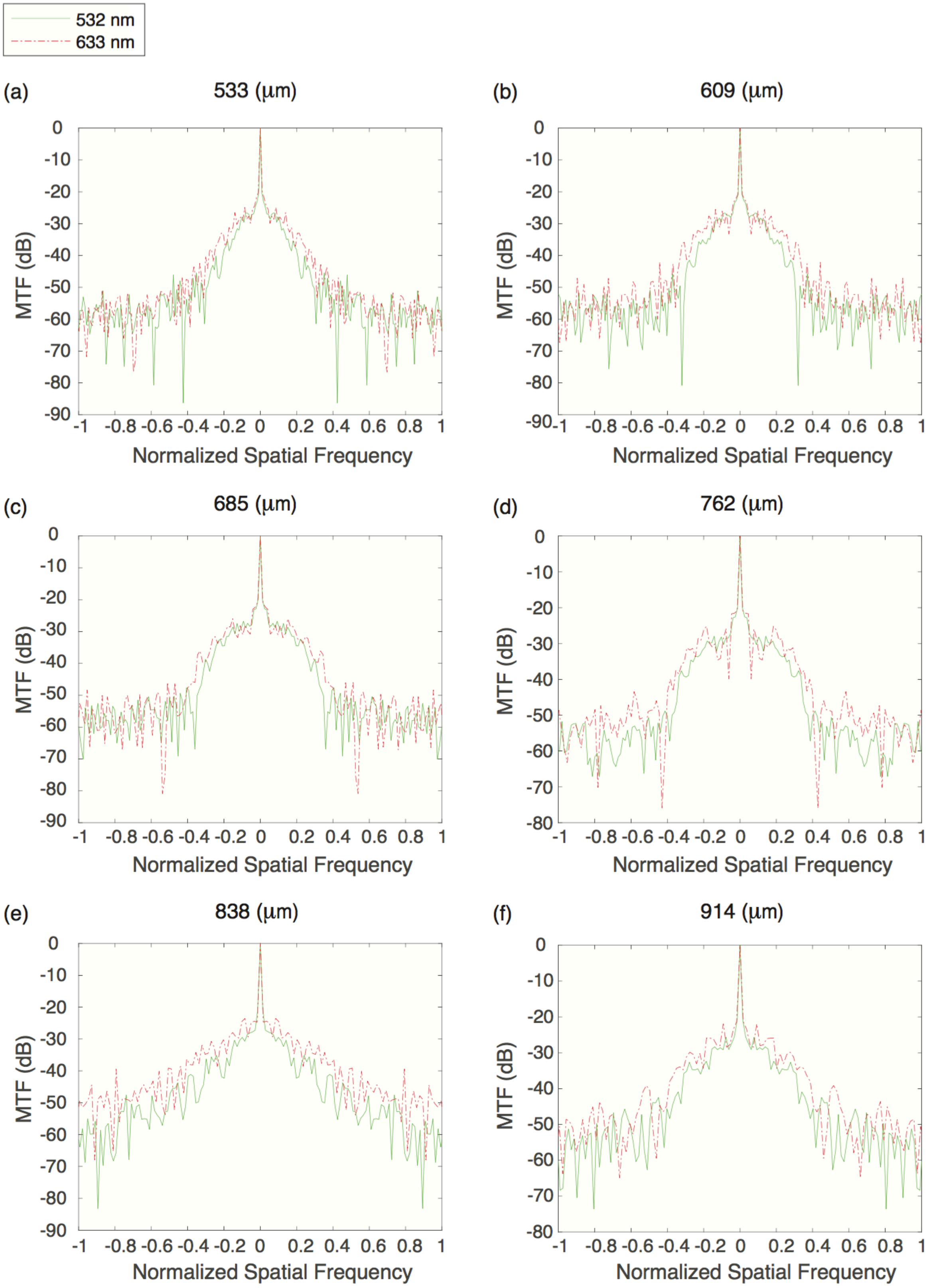
532 nm
633 nm
(a)
533 (μm)
(b)
609 (μm)
(c)
685 (μm)
(d)
762 (μm)
(e)
838 (μm)
(f)
914 (μm)
MTF (dB)
Normalized Spatial Frequency
0
-10
-20
-30
-40
-50
-60
-70
-80
-90
-1
-0.8
-0.6
-0.4
-0.2
0
0.2
0.4
0.6
0.8
1

Fig. S8: Modulation transfer functions of the cubic element. (a)-(f) show 1D slices of the MTF of the cubic element for a range of over 300 μm plotted against normalized spatial frequency for both red and green illumination. The MTFs for green (532 nm) and red (633 nm) are shown in solid and dotted lines respectively.

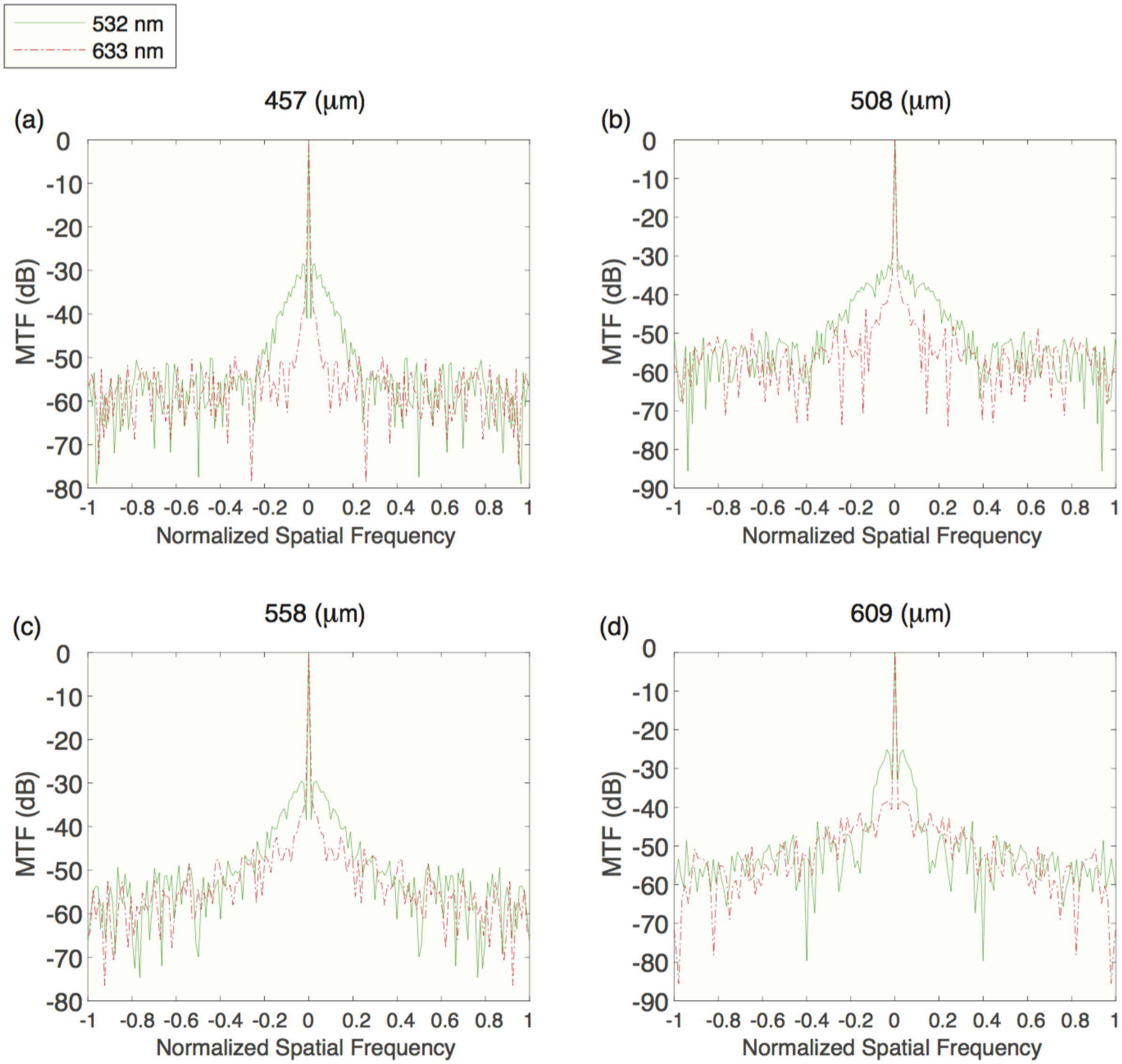

Fig. S9: Modulation transfer function of the 500 μm quadratic metasurface lens. (a)-(d) show 1D slices of the MTF of the quadratic element for a range of 150 μm plotted against normalized spatial frequency for both red and green illumination. The MTFs for green (532 nm) and red (633 nm) are shown in solid and dotted lines respectively.

1 Dowski, E. R. & Cathey, W. T. Extended depth of field through wave-front coding. *Appl. Opt.* **34**, 1859-1866, doi:10.1364/AO.34.001859 (1995).
2 Wach, H. B., Dowski, E. R. & Cathey, W. T. Control of chromatic focal shift through wave-front coding. *Appl. Opt.* **37**, 5359-5367, doi:10.1364/AO.37.005359 (1998).
3 Bradburn, S., Cathey, W. T. & Dowski, E. R. Realizations of focus invariance in optical–digital systems with wave-front coding. *Appl. Opt.* **36**, 9157-9166, doi:10.1364/AO.36.009157 (1997).
